# ATP/IL‐33‐triggered hyperactivation of mast cells results in an amplified production of pro‐inflammatory cytokines and eicosanoids

**DOI:** 10.1111/imm.13386

**Published:** 2021-06-30

**Authors:** Paul M. Jordan, Nico Andreas, Marco Groth, Philine Wegner, Franziska Weber, Ute Jäger, Claudia Küchler, Oliver Werz, Edgar Serfling, Thomas Kamradt, Anne Dudeck, Sebastian Drube

**Affiliations:** ^1^ Department of Pharmaceutical/Medicinal Chemistry Institute of Pharmacy Friedrich Schiller University Jena Jena Germany; ^2^ Institute of Immunology Jena University Hospital Jena Germany; ^3^ CF DNA Sequencing Fritz Lipmann Institute Jena Germany; ^4^ Department of Molecular Pathology Institute of Pathology University Würzburg Würzburg Germany; ^5^ Institute for Molecular and Clinical Immunology Otto‐von‐Guericke Universität Magdeburg Magdeburg Germany; ^6^ Health Campus Immunology, Infectiology and Inflammation Medical Faculty Otto‐von‐Guericke Universität Magdeburg Magdeburg Germany

**Keywords:** ATP, co‐sensing, hyperactivation, IL‐33, mast cells

## Abstract

IL‐33 and ATP are alarmins, which are released upon damage of cellular barriers or are actively secreted upon cell stress. Due to high‐density expression of the IL‐33 receptor T1/ST2 (IL‐33R), and the ATP receptor P2X7, mast cells (MCs) are one of the first highly sensitive sentinels recognizing released IL‐33 or ATP in damaged peripheral tissues. Whereas IL‐33 induces the MyD88‐dependent activation of the TAK1‐IKK2‐NF‐κB signalling, ATP induces the Ca^2+^‐dependent activation of NFAT. Thereby, each signal alone only induces a moderate production of pro‐inflammatory cytokines and lipid mediators (LMs). However, MCs, which simultaneously sense (co‐sensing) IL‐33 and ATP, display an enhanced and prolonged activation of the TAK1‐IKK2‐NF‐κB signalling pathway. This resulted in a massive production of pro‐inflammatory cytokines such as IL‐2, IL‐4, IL‐6 and GM‐CSF as well as of arachidonic acid‐derived cyclooxygenase (COX)‐mediated pro‐inflammatory prostaglandins (PGs) and thromboxanes (TXs), hallmarks of strong MC activation. Collectively, these data show that co‐sensing of ATP and IL‐33 results in hyperactivation of MCs, which resembles to MC activation induced by IgE‐mediated crosslinking of the FcεRI. Therefore, the IL‐33/IL‐33R and/or the ATP/P2X7 signalling axis are attractive targets for therapeutical intervention of diseases associated with the loss of integrity of cellular barriers such as allergic and infectious respiratory reactions.

## Graphical abstract

Co‐sensing of ATP and IL‐33 triggers hyperactivation of mast cells resulting in an amplified production of pro‐inflammatory cytokines and eicosanoids. Imagewas created with BioRender.com.

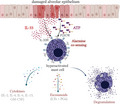

**Abbreviations**
5‐LOX: 5‐lipoxygenase
ATP: adenosine triphosphate
BMMCs: bone marrow–derived MC
Ca^2+^
: Calcium
CaN: calcineurin
CHS: contact hypersensitivity
COVID‐19: corona virus disease
COX‐1/2: cyclooxygenases‐1 and ‐2
IL‐33: interleukin‐33
LMs: lipid mediators
MAPKs: mitogen‐activated protein kinases
MC: mast cell
NFAT: nuclear factor of activated T‐cells
NF‐κB: nuclear factor‐κB
PCMCs: peritoneal cavity–derived MCs
PGs: prostaglandins
TXs: thromboxanes

## INTRODUCTION

In peripheral tissues, MCs are key effector cells in type I hypersensitivity reactions [1]. In detail, MC stimulation by antigen bound to IgE and the consequent FcεRI receptor crosslinking results in a Ca^2+^‐dependent activation of the nuclear factor of activated T cells (NFAT), mitogen‐activated protein kinases (MAPKs) and nuclear factor‐κB (NF‐κB) [2]. In turn, this leads to MC degranulation, cytokine production [2], and to eicosanoid formation by activation of cyclooxygenase‐1 and cyclooxygenase‐2 (COX‐1/2) and of 5‐lipoxygenase (5‐LOX) [3] culminating in hypersensitivity reactions. MCs have also been shown to contribute to tissue integrity by expressing receptors sensing cell stress such as T1/ST2 (IL‐33R) [4, 5], and the ATP receptor P2X7. IL‐33 activates the MyD88‐TAK1‐IKK2‐p65 signalling pathway and MAPKs, resulting in cytokine biosynthesis [5], but not degranulation [6]. In contrast to this, binding of ATP to the Ca^2+^ channel P2X7 [7] activates NFAT and MAPKs, which are crucial for the production of cytokines and degranulation [8, 9] as well as for the biosynthesis of 5‐LOX‐derived leukotrienes (LTs) [10]. Furthermore, ATP induces the P2X7‐dependent activation of the NLRP3 inflammasome, which results in cleavage of pro‐IL‐1β and pro‐IL‐18 and thereby, in the release of mature IL‐1β and IL‐18 [11]. Of note, IL‐33 and ATP are alarmins that are released by damage of the integrity of cellular barriers or are actively secreted upon cell stress [9]. Due to the high expression of the IL‐33R and P2X7 [7], MCs are one of the first cellular sensors for IL‐33 and ATP in peripheral tissues [12]. Notably, co‐sensing of ATP and IL‐33 by MCs is required to initiate the full vascular response resulting in contact hypersensitivity reactions (CHS) *in vivo* [12]. Here, we identified the cellular mechanism essential for MC‐dependent CHS reactions. We show that ATP/IL‐33 co‐sensing by MCs results in a persistent activation of the TAK1‐IKK2‐p65 signalling pathway resulting in the potentiated production of IL‐2, IL‐4, IL‐6, GM‐CSF and TNFα as well as of pro‐inflammatory LMs. The facts that the levels of IL‐33 and ATP are elevated in patients with COVID‐19 and that the activation status of MCs correlates with the severity of COVID‐19 [13, 14] indicate that the principle of ATP/IL‐33 co‐sensing might also be relevant for the development of severe COVID‐19. These data in combination with our recent observation that MCs can degranulate directionally into the bloodstream [15] suggest that MCs co‐activated by ATP and IL‐33 might be an attractive therapeutic target to dampen cytokine storm syndromes, shock symptoms or allergic reactions.

## RESULTS

### ATP as a strong inducer of MC degranulation

**FIGURE 1:**
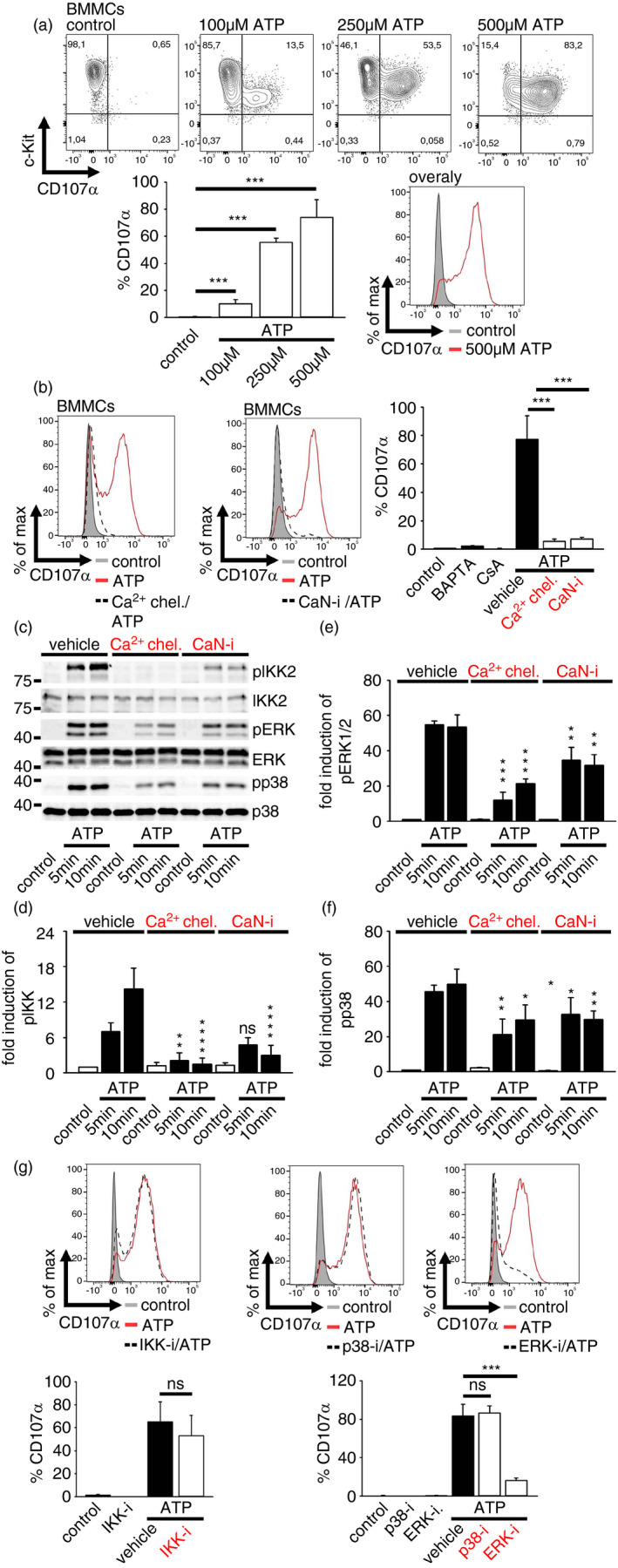
ATP‐induced MC degranulation. (a) BMMCs were stimulated with ATP (30 min) as indicated and were stained for CD107α and c‐Kit. Shown are contour blots or an overlap histogram as well as the summary of *n* = 3 biological replicates. Shown is the mean ± SD; paired *t*‐test; ***p ≤ 0·001. (b) Prior to ATP stimulation, BMMCs were pre‐treated with the indicated inhibitors (Ca^2+^ chel. = Ca^2+^ chelator; CaN‐i = Calcineurin inhibitor) for 30 min. Shown are histograms or the summary of *n* = 3 biological replicates as the means ± SD; paired *t*‐test; ***p ≤ 0·001. (c–f) Cell lysates were analysed by Western blotting. (d–f) Shown are the densitometric analysis of *n* = 3 biological replicates as the means ± SD; paired *t*‐test against the appropriated vehicle/ATP stimulation; ns: not significant, *p ≤ 0·05, **p ≤ 0·01, ***p ≤ 0·001. The molecular weight is shown on the left side of the Western blots. (g) BMMCs were pre‐treated with the indicated inhibitors (IKK‐i = IKK‐inhibitor; p38‐i = p38 inhibitor; ERK‐i = ERK‐inhibitor) for 30 min and were stimulated with ATP for 30 min. Shown are histograms and the summary of *n* = 3 biological replicates as the mean ± SD; paired *t*‐test; ns, not significant; ***p ≤ 0·001

Recently, we found that IL‐33 induces a MC degranulation–dependent skin inflammation [6]. However, IL‐33 alone does not induce MC degranulation *in vitro* indicating that an additional stimulus may co‐activate MCs for the induction of degranulation *in vivo* [6]. Notably, ATP is required to induce the IL‐33‐dependent vascular response to contact allergens, indicating that ATP/IL‐33 co‐sensing mediates a strong MC activation characterized by degranulation [12]. We first determined the ATP‐induced signalling and the resulting degranulation. To investigate the general effects on MCs and to avoid cell‐specific effects, we used bone marrow‐derived MC (BMMCs) as well as peritoneal cavity‐derived MCs (PCMCs). Indeed, high levels of ATP induced a massive degranulation in MCs (shown by CD107α surface expression [16]) (Figure 1a; Figure S1a), which was accompanied by c‐Kit downregulation in BMMCs and PCMCs (Figure S1b). These characteristics were inhibited by the selective P2X7 inhibitor A438079 (Figure S1c), demonstrating that the ATP/P2X7 axis induced the formation of highly activated c‐Kit^low^/CD107α^high^ degranulating MCs. MC degranulation depends on Ca^2+^ and CaN [17]. Confirming these data, ATP induced a Ca^2+^ influx, which was dependent on P2X7 (Figure S1d), and subsequently resulted in a Ca^2+^/CaN‐mediated degranulation in BMMCs and PCMCs (Figure 1b; Figure S1e). Furthermore, ATP did not activate TAK1 but activated IKK2, p38 and ERK1/2 (Figure S1f–j), and in line with the degranulation response, the activation of these signalling molecules was dependent on the Ca^2+^/CaN signalling module (Figure 1c–f). However, only ERK1/2 but not IKKs or p38 mediated the ATP‐induced degranulation in BMMCs and PCMCs (Figure 1g; Figure S1k). These findings show that ATP caused a strong Ca^2+^/CaN‐ERK1/2‐dependent MC degranulation.

### ATP/IL‐33 co‐sensing boosted the induced cytokine responses

**FIGURE 2:**
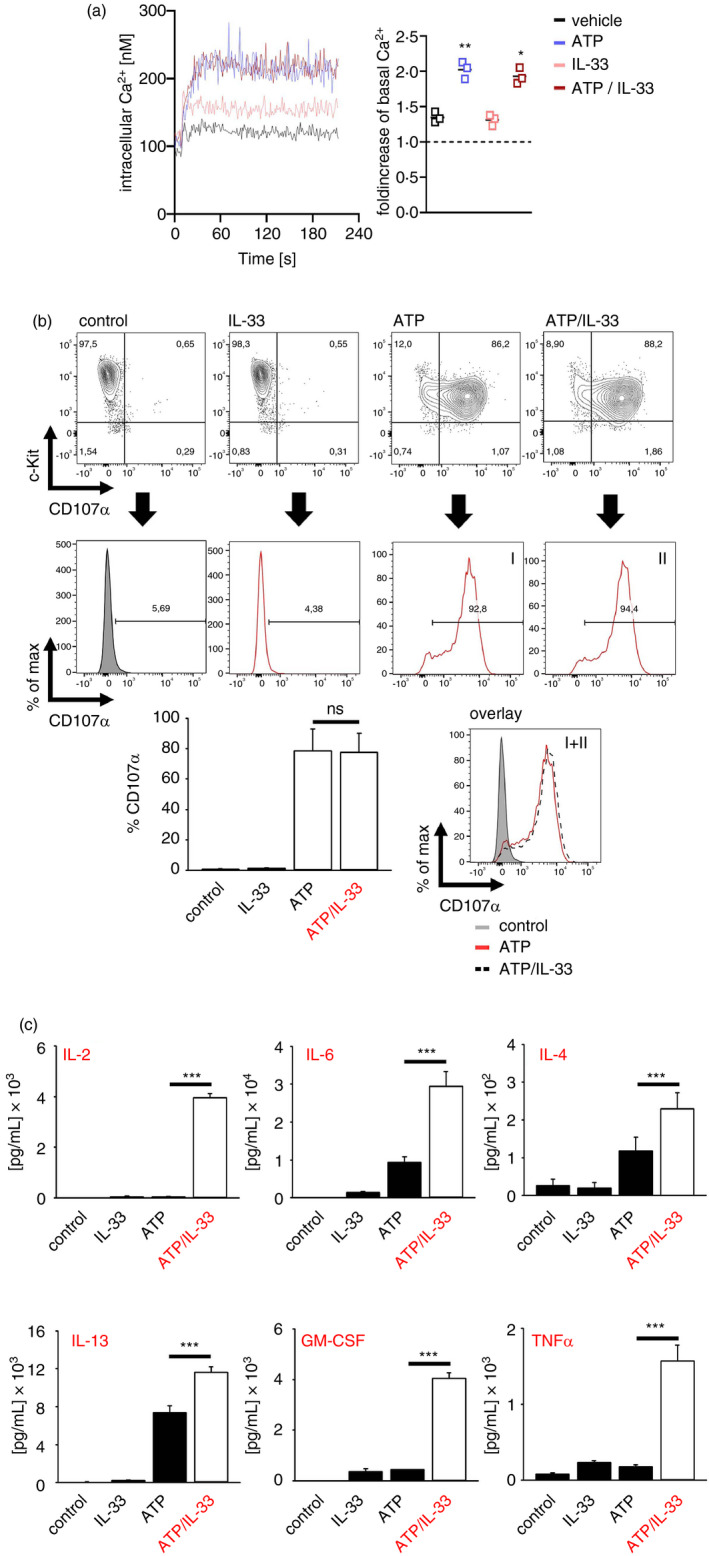
Co‐sensing of ATP and IL‐33 potentiates the resulting cytokine production. (a) Ca^2+^ influx was measured from BMMCs stimulated with ATP, IL‐33 or both together. Shown are measured [Ca^2+^]_I_ after indicated time points (left panel) and the single values as fold increase of the mean of [Ca^2+^]_I_ after stimulation against the mean of basal [Ca^2+^]_I_. Shown are the summary *n* = 3 biological replicates, paired *t*‐test against vehicle; *p ≤ 0·05, **p ≤ 0·01. (b) BMMCs were stimulated with IL‐33, ATP or both together for 30 min. Shown are contour blots or the summary of *n* = 3 biological replicates as the mean ± SD; paired *t*‐test against ATP stimulation; ns: not significant. (c) Wt BMMCs were stimulated with IL‐33, ATP or both together for 24 h. Supernatants were analysed by cytokine ELISAs. Shown is the mean ± SD from *n* = 3 ELISA experiments; paired *t*‐test; ***p ≤ 0·001

Next, we determined the influence of IL‐33 on ATP‐induced MC effector functions. When we evaluated whether the ATP‐induced degranulation is modified by IL‐33, we could neither detect an alteration of the ATP‐induced Ca^2+^ mobilization **(**Figure 2a) nor of MC degranulation (Figure 2b). Therefore, rapidly triggered MC effector functions in response to ATP, such as the degranulation‐mediated release of preformed mediators, are not modulated by IL‐33. However, the rapid MC degranulation is followed by a later *de novo* production of cytokines. Stimulation of MCs with ATP or IL‐33 alone induced a moderate production of IL‐2, IL‐4, IL‐6, IL‐13, GM‐CSF and TNFα (Figure 2c). Surprisingly, co‐stimulation with ATP and IL‐33 potentiated the production of IL‐2, IL‐4, IL‐6, IL‐13, GM‐CSF and TNFα compared to either stimuli alone (Figure 2c). Consequently, our data show that concomitant sensing of ATP and IL‐33 culminates in a MC hyperactivation that boosts the cytokine biosynthesis.

### ATP/IL‐33 co‐sensing prolonged the activation of p65/RelA

**FIGURE 3:**
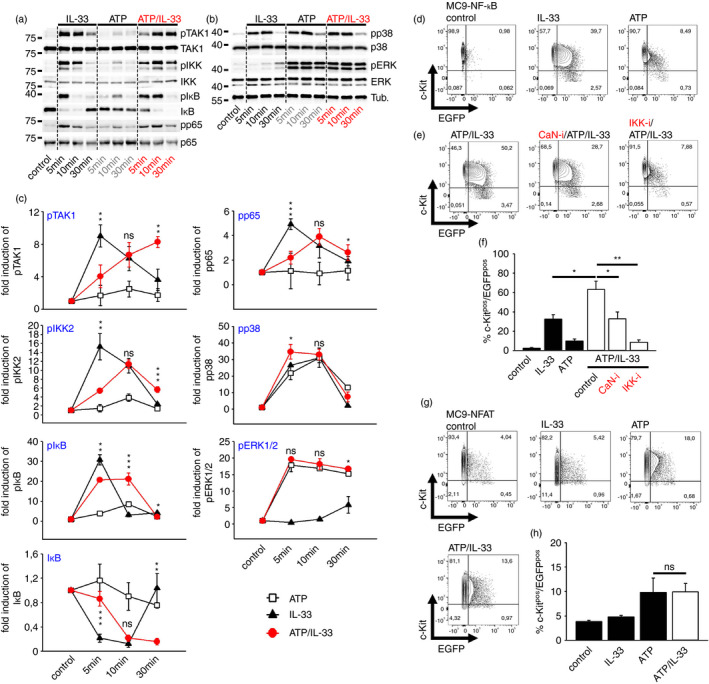
Concomitant ATP/IL‐33 sensing results in a persistent activation of the TAK1‐IKK2‐p65/RelA signalling pathway. (a, b) BMMCs were stimulated with IL‐33 or ATP or both together as indicated. (c) Shown is densitometric analysis of Western blots of *n* = 3 biological replicates as the mean ± SD; paired *t*‐test against IL‐33 stimulations (except of pERK1/2 which was calculated against the ATP stimulation); ns: not significant, *p ≤ 0·01, **p ≤ 0·05, ***p ≤ 0·001. The molecular weight is indicated on the left side of the Western blots. (d) NF‐κB‐MC/9 reporter MCs were stimulated with IL‐33 or ATP alone for 8 h. (e) NF‐κB‐MC/9 reporter MCs were stimulated with IL‐33 and ATP together for 8h. Prior to stimulation cells were pre‐treated with the indicated inhibitors (CaN‐i = Calcineurin inhibitor; IKKi = IKK inhibitor) for 30 min. Shown are representative experiments (d, e) and the summary of *n* = 3 technical replicates (f). ns: not significant, *p ≤ 0·01, **p ≤ 0·05. (g) NFAT‐MC/9 cells were stimulated with either IL‐33 or ATP or co‐stimulated with both for 8 h. Shown are representative experiments (g) and the summary of *n* = 3 technical replicates (h). ns, not significant

Given the excessive cytokine production in MCs hyperactivated by ATP/IL‐33 co‐sensing, we investigated how IL‐33 interferes with the ATP‐induced and Ca^2+^/CaN‐dependent activation of IKK1/2, ERK1/2 and p38. Confirming prior studies [5, 6], IL‐33 alone induced a strong but transient activation of the TAK1‐IKK2/IκB signalling module (Figure 3a–c). In contrast to this, ATP did not efficiently activate TAK1, but slightly activated IKK2 (Figure 3a–c). However, co‐stimulation with ATP and IL‐33 resulted in a delayed activation of the TAK1‐IKK2/IκB signalling module (Figure 3a,c), which was surprisingly sustained beyond 60 min compared to single stimulation with IL‐33 or ATP alone (Figure S2a,b). Next, we investigated the influence of ATP/IL‐33 co‐stimulation on the activation of p65/RelA, p38 and ERK1/2 the most essential signalling pathways for IL‐33‐ and/ or ATP‐induced effector functions [6, 18, 19]. We found that the persistently active TAK1‐IKK2/IκBα signalling pathway resulted in a delayed and prolonged activation of p65/RelA, whereas the activation of p38 or ERK1/2 was only slightly affected (Figure 3a–c; Figure S2a,b). Importantly, the overall amplified activation of p65/RelA by ATP/IL‐33 co‐sensing was confirmed in MC/9‐NF‐κB‐eGFP reporter MCs by the detection of an enhanced eGFP signal. Compared to ATP alone, IL‐33 efficiently induced the expression of eGFP demonstrating p65/RelA activation (Figure 3d,f). Co‐stimulation with ATP and IL‐33 further increased the p65/RelA activation, which was mediated by CaN and IKKs (Figure 3e,f). In contrast to this, IL‐33 neither induced nor modulated the ATP‐induced activation of NFAT in MC/9‐NFAT‐eGFP reporter cells (Figure 3g,h). Collectively, these data indicate that ATP, likely via the induced Ca^2+^ influx, supported the IL‐33‐induced signalling pathways by prolonging the activation of TAK1 and thus of the IKK2/IκB‐p65/RelA signalling pathway, while NFAT remained unaffected.

### The concerted activation of p65/RelA and NFATc2 enhanced the production of cytokines

**FIGURE 4:**
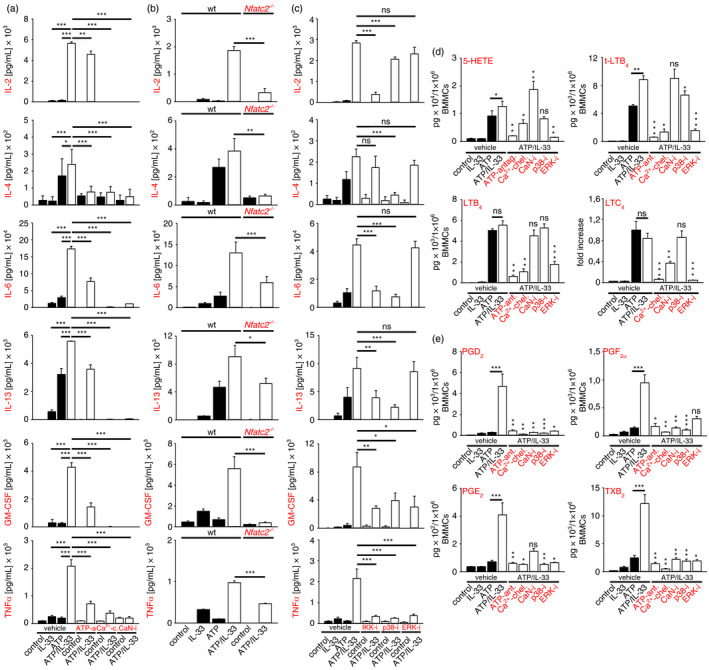
The Ca^2+^/CaN signalling module and IKKs are essential for the potentiated cytokine and eicosanoid response. (a–c) Wt (a–c) or wt and *Nfatc2* ^−/−^ (b) BMMCs were stimulated with IL‐33 or ATP alone (black bars) or both together (white bars) for 24 h. (a, c) To block ATP and Ca^2+^ or to inhibit CaN, IKKs, p38 or ERK1/2, prior to stimulation, BMMCs were pretreated with the respective agents or inhibitors (ATP‐a = ATP‐antagonist; Ca^2+^‐c = Ca^2+^ chelator; IKK‐i = IKK‐inhibitor; p38‐i = p38 inhibitor; ERK‐i = ERK inhibitor) for 30 min. (a‐c) Supernatants were analysed for cytokines by ELISAs. (a, c) Shown are the means ± SD from *n* = 3 biological replicates; paired *t*‐test between the indicated groups; ns: not significant, *p ≤ 0·05, **p ≤ 0·01, ***p ≤ 0·001. (b) Shown are the means ± SD from *n* = 3 biological replicates from BMMCs of wt and *Nfatc2* ^−/−^ mice; paired *t*‐test; *p ≤ 0·05, **p ≤ 0·01, ***p ≤ 0·001. (d, e) Shown is the eicosanoid release. To block ATP and Ca^2+^ or to inhibit CaN, p38 or ERK1/2 BMMCs were pre‐treated with the respective agents or inhibitors (ATP‐a = ATP‐antagonist; Ca^2+^‐c = Ca^2+^ chelator; p38‐i = p38 inhibitor; ERK‐i = ERK inhibitor) for 30 min prior to stimulation with IL‐33 or ATP or both together for 24 h. Except of LTC_4_, generated eicosanoids were extracted and analysed by UPLC‐MS‐MS. LTC_4_ was analysed by a LTC_4_‐specific ELISA. Shown in the fold increased compared to the pg‐values of ATP/IL‐33 stimulations, which were set as 1. Data are the means ± SEM, from *n* = 3 biological replicates; paired *t*‐test between ATP‐single (black) and ATP/IL‐33 co‐stimulated BMMCs (white bars) or between vehicle/ATP/IL‐33‐ and inhibitor/ATP/IL‐33‐stimulated BMMCs (white bars); ns: not significant, *p ≤ 0·05, **p ≤ 0·01, ***p ≤ 0·001

IL‐33 activates the MyD88‐TAK1/IKK2‐p65/RelA, but not the Ca^2+^/CaN‐NFAT signalling pathway and induces the production of IL‐2, IL‐6, IL‐13 and GM‐CSF in MCs [18, 19]. In contrast to this, the ATP‐induced cytokine production strongly depended on the Ca^2+^/CaN‐signalling module in conjunction with NFATc2, IKKs, p38 and ERK1/2 (Figure S3a–c). While the co‐stimulatory effect of ATP/IL‐33 was fully dependent on the IL‐33R (Figure S4a), it was only partially reduced by the selective P2X7 inhibitor A438079 (Figure 4a), which suggested that other ATP receptors may have compensated the P2X7 inhibition. In line with ATP single stimulation, all cytokines produced upon ATP/IL‐33 co‐stimulation were controlled by the Ca^2+^/CaN signalling module (Figure 4a) underpinning its key function in activated MCs. However, in ATP/IL‐33‐co‐activated MCs, NFATc2 substantially contributed to the IL‐6 production, which was in contrast to stimulation with ATP alone (Figure 4b; Figure S3b). This demonstrated that ATP/IL‐33 co‐stimulation broadens the spectrum of NFAT to mediate the production of an increased range of cytokines. Another central signalling node that is targeted by IL‐33 or ATP are IKKs, which are essentially involved in the activation of p65/RelA and in the resulting cytokine production [5]. Similar to their role in ATP‐single stimulation, IKKs are essential for the ATP/IL‐33 co‐signalling‐induced production of all analysed cytokines except IL‐4 (Figure 4c). Whereas p38 is essential, ERK1/2 was dispensable for most of the cytokines produced in ATP/IL‐33‐co‐stimulated MCs (Figure 4c). Underpinning the critical role of the Ca^2+^/CaN signalling network and of p38 in the potentiated production of IL‐2, IL‐4, IL‐6, IL‐13 and GM‐CSF in response to ATP/IL‐33 co‐activation in all MCs, these data were also confirmed in PCMCs (Figure S4b). Collectively, these data demonstrate that the Ca^2+^/CaN‐NFATc2 and the TAK1‐IKK2‐p65 signalling pathway in conjunction with p38 triggers a massive cytokine response upon ATP/IL‐33 co‐sensing.

### Beyond cytokines, co‐sensing of ATP and IL‐33 boosts the activation of COX1/2

MCs in general are major sources for LMs like leukotrienes (LTs) and prostaglandins (PGs) [20, 21]. Reflecting the induced cytokine responses, ATP/IL‐33 co‐sensing is required to induce effective 5‐LOX‐ and COX1/2‐mediated product formation (Figure S5a). IL‐33 alone did not activate 5‐LOX, whereas stimulation with ATP induced the production of LTB_4_, t‐LTB_4_ and 5‐HETE (Figure S5a), via the P2X7‐Ca^2+^‐ERK1/2‐5‐LOX signalling pathway, independently from CaN, p38 (Figure S5b) and NFATc2 (Figure S5c). Furthermore, IL‐33 or ATP alone only slightly induced the activation of COX‐1/2, which resulted in the production of low levels of PGs and TXB_2_ (Figure S5a). In contrast to this, ATP/IL‐33 co‐sensing increased the production of 5‐HETE and 6‐trans LTB_4_ (t‐LTB_4_) but not of LTB_4_. Thereby, the production of 5‐HETE, t‐LTB_4_ and LTB_4_ was mediated via the P2X7‐Ca^2+^‐ERK1/2 signalling pathway but independent from CaN, p38 (Figure 4d) or NFATc2 (Figure S5d). We also investigated the production of cysteinyl LTs such as LTC_4_ a major mediator of MC effector functions. The analysis of LTC_4_ by the current UPLC‐MS‐MS method is not suitable due to the different chemical structures of glutathione‐conjugated LM; therefore, we used a LTC_4_‐specific ELISA. In line with the unaltered LTB_4_ levels, ATP/IL‐33 co‐sensing did not further increase the production of LTC_4_, which also was produced in dependency of a Ca^2+^‐ERK1/2 signalling pathway (Figure 4d).

Importantly, ATP/IL‐33 co‐sensing resulted in a massively induced production of PGE_2_, PGD_2_, PGF_2α_ and TXB_2_ (Figure 4e). However, contrasting the mechanism, which increases the cytokine responses, the P2X7‐Ca^2+^/CaN signalling module, p38 as well as ERK1/2 (Figure 4e) but not NFAT (Figure S5e), mediated the production of these LMs. Except for the production of PGE_2_, which was only slightly induced in response to concomitant ATP/IL‐33 sensing, the importance of the Ca^2+^‐ERK1/2 signalling pathway in the induction of 5‐LOX or COX1/2 products could be confirmed in PCMCs (Figure S6a–c). Notably, these data show that in response to ATP/IL‐33 co‐sensing MCs produce massively increased amounts of pro‐inflammatory LMs.

### ATP/IL‐33 co‐sensing: a principle relevant for COVID‐19 pathogenesis?

**FIGURE 5:**
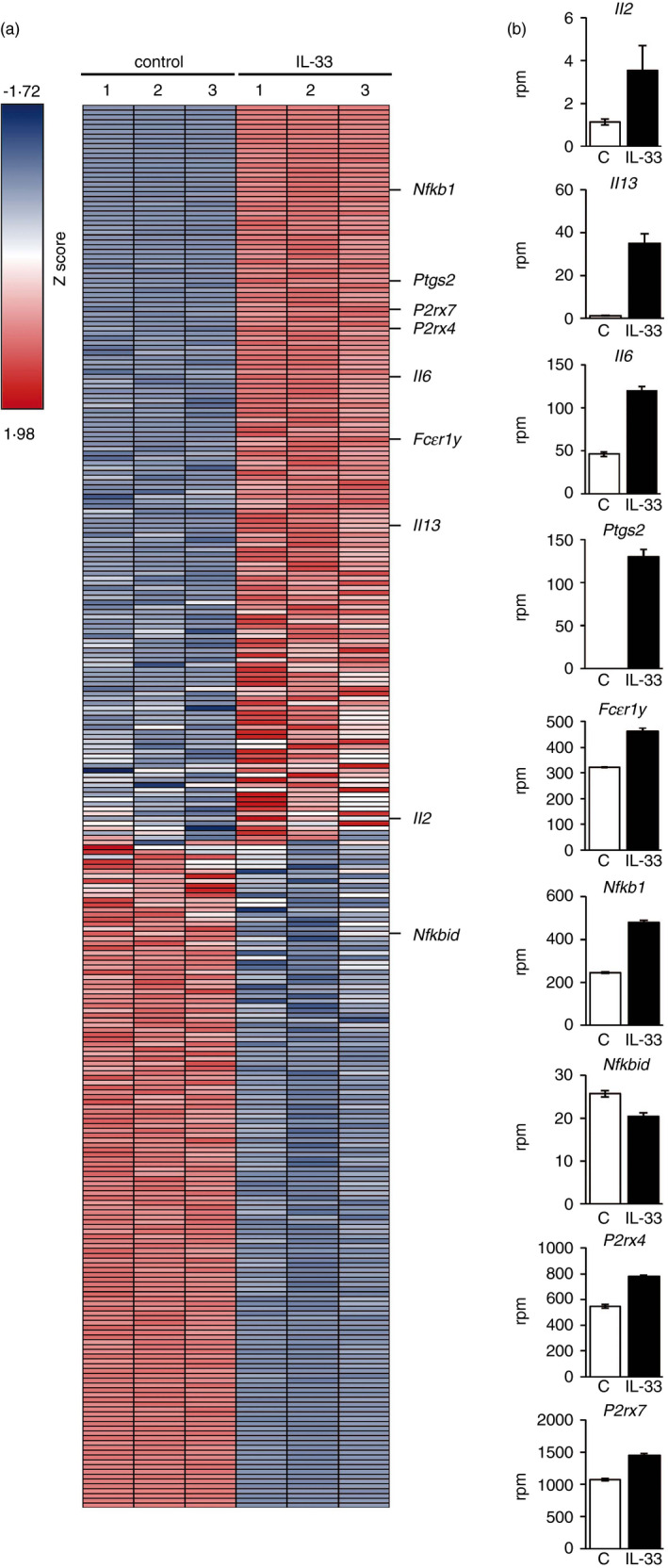
IL‐33 upregulates the expression purinergic receptors. (a) DEGs of IL‐33‐stimulated (for 24 h) MCs (*n* = 3 biological replicates for control and IL‐33 stimulation) were filtered for genes representing the MPO cluster ‘abnormal innate immunity’ [42] and their expression is shown as *Z*‐score in the heat map. (b) Expression levels of selected genes are shown in separate diagrams as reads per million (rpm) genes detected. In the bar diagrams, average ±SEMs are shown

In lung tissues of patients with severe COVID‐19, the levels of ATP and IL‐33 are strongly increased, concurrent with the presence of highly activated MCs [13, 14, 22, 23, 24]. This suggests that MCs are strongly co‐activated by the released ATP/IL‐33 in the airways of COVID‐19 patients. To investigate this possibility, we used the NGS data set generated from Winkler *et al*. [25] to search for upregulated genes that are highly expressed in MCs [26] and in total lung tissue of transgenic hACE2 mice upon SARS‐CoV‐2 infection. Indeed, we found 12 genes that represent MCs [26] among genes upregulated in lung tissue from transgenic hACE2 infected with SARS‐CoV‐2 [25] (Figure S7a). Because IL‐33 sensitizes the local environment and also MCs towards tissue‐damaging events [2, 12] and we showed that the IL‐33‐induced NF‐κB activation dominates in ATP/IL‐33‐co‐activated MCs, we compared genes differentially expressed in IL‐33‐activated MCs with genes differentially expressed in lung tissue of SARS‐CoV‐2‐infected hACE2 mice [25]. We identified 2410 intersecting genes, which were analysed with the help of WebGestalt [27, 28, 29, 30]. A total of 2386 of these genes could be mapped to entrez gene and were subsequently clustered by an overrepresentation analysis (ORA) via the mammalian phenotype database (The Jackson Laboratories). Gene clustering with a false discovery rate (FDR) of less than 10^−14^ as calculated by Benjamini and Hochberg's procedure resulted in 11 categories (Figure S7b). Among them, the category ‘abnormal innate immunity’ showed the second‐highest coverage ratio (Figure S7b). We filtered the differentially expressed genes in IL‐33‐stimulated MCs for genes constituting the cluster ‘abnormal innate immunity’. Of note, not all differentially expressed genes associated with this cluster were upregulated in MCs upon IL‐33 stimulation (Figure 5a). Along with upregulated transcripts of IL‐2 and IL‐13, we found upregulated IL‐6 transcripts in MCs upon IL‐33 stimulation validating successful MC activation [6, 19] (Figure 5a). Reflecting the increased LM production upon ATP/IL‐33 co‐sensing, the *Ptgs2* transcripts were massively upregulated upon IL‐33 stimulation (Figure 5a). While the *FcεrIα* was not found among the differentially expressed genes (data not shown), we detected increased transcript levels of *Fcεr1γ*. Moreover, the transcript level of the canonical NF‐κB subunit *Nfkb1* (p50) (Figure 5b) was upregulated, whereas the transcript levels of *Nfκbid* (the negative regulator of NF‐κB) was downregulated in IL‐33‐stimulated MCs (Figure 5b). Importantly, IL‐33 upregulated the ATP receptors P2X4 and P2X7 (Figure 5b). This in line with the fact that ATP receptors were also upregulated in lungs of SARS‐CoV‐2‐infected hACE2 mice [25], this demonstrates that IL‐33 sensitizes the local environment and also MCs [2, 12] to released ATP. These data point out a potential cross talk between activated IL‐33R and P2X7 and show that of ATP/IL‐33 co‐sensing by MCs might be relevant for COVID‐19 progression.

## DISCUSSION

IL‐33 is expressed in endothelial and epithelial cells and is actively or passively released upon cell stress or cell damage, respectively. In previous studies, we showed that contact hypersensitivity (CHS) reactions in the skin, which are triggered upon induction of stress and damage of epidermal cells, depend on simultaneous sensing of IL‐33 and ATP by MCs and, subsequently, results in a MC‐dependent recruitment of neutrophils [6, 12]. Inactivation of either of these signals alone on MCs blocked neutrophil recruitment and thereby, the resulting CHS reaction [6, 12]. Here, we identified the mechanism in MCs behind the strong and transient MC‐dependent CHS reaction. Confirming the principle of ATP/IL‐33 co‐sensing by MCs *in vivo* [12], stimulation with ATP or IL‐33 alone resulted only in a weak MC activation *in vitro*. Whereas ATP alone resulted in degranulation and a moderate cytokine production via a Ca^2+^/CaN‐signalling network, IL‐33 alone did not induce degranulation but initiated a MyD88‐dependent moderate cytokine production [5]. In contrast to the weak MC activation induced by either signal alone, ATP/IL‐33 co‐sensing resulted in formation of hyperactivated MCs (Figure S8). These MCs did strongly degranulate and boosted the production of pro‐inflammatory cytokines such as IL‐2, IL‐4, IL‐6, GM‐CSF and TNFα, and pro‐inflammatory arachidonic acid‐derived LMs (Figure S8). Herein, the initial transient TAK1 activation by IL‐33 is converted into a persistent TAK1 activation by co‐stimulation with ATP. This in turn prolonged the activity of the IKK2/IκB‐p65/RelA signalling pathway, which is essential to induce the functional interaction between p65/RelA, NFATc2 and p38, and ultimately resulted in a potentiated cytokine production (Figure S9). Thereby, we hypothesize that the prolonged activation of p65/RelA supports NFAT‐ and MAP‐kinase‐dependent effector functions, which might be mediated by direct interactions between NFAT, NF‐κB and/or MAP‐kinases as recently reported [31, 32, 33] (Figure S9). This in turn increases the functionality of these transcription factors and potentiates the resulting MC effector functions. Notably, the phenotype of hyperactivated MC and the signalling mechanisms induced by concomitant ATP/IL‐33‐sensing are comparable to the activation modes induced by FcεRI stimulation [2]. Consequently, the innate immune system possesses two mechanisms, which result in strong MC activation: (i) the fast but antigen‐independent MC hyperactivation stimulated via liberated alarmins such as ATP and IL‐33 and (ii) the delayed and IgE‐dependent MC activation depending on the adaptive immune system. Interestingly, the level of ATP and IL‐33 is elevated in patients with severe COVID‐19 [13, 22, 23], which might be explained by the massive destruction of lung tissues in responses to SARS‐CoV‐2 infection. Given the fact that MCs are tissue‐resident myeloid cells with a high and stable expression of the IL‐33R and of P2X7 in lung tissues, we hypothesize that MCs sensitively recognize SARS‐CoV‐2‐induced damage of epithelial cells by sensing liberated IL‐33 and ATP. This might result in the formation of activated MCs, which were found in COVID‐19 patients [24]. This hypothesis is suggested by the fact that we detected upregulated MC genes in SARS‐CoV‐2‐infected hACE2 mice [25] as well as upregulated purinergic receptors, which we also detected to be upregulated by IL‐33 on MCs. Interestingly, the pathogenesis of COVID‐19 is characterized by recruitment and local activation of neutrophils, monocytes/macrophages and T cells and thereby mirrors the mechanisms found in MC‐dependent CHS reactions [6, 12].

Intriguingly, we recently found that upon CHS‐induced skin inflammation, the perivascular MCs (activated by IL‐33/ATP co‐sensing [6, 12]) have the capacity to penetrate the endothelial barriers and to degranulate directionally into blood vessels [15]. Therefore, we hypothesize that upon hyperactivation, this MC capacity might contribute to CHS reactions and also to the COVID‐19‐associated cytokine and eicosanoid storm. Connecting all these facts with our data presented here, we suggest that the principle of ATP/IL‐33‐hyperactivated MCs is relevant for the development of cytokine storm syndromes, shock symptoms or allergic reactions. Consequently, we conclude that therapeutic strategies, which block the IL‐33/IL‐33R or the ATP/P2X7 signalling axis may represent a potential option to treat hyperinflammatory diseases.

## MATERIALS AND METHODS

### Mice

WT mice were maintained at the Animal Research Facility of the Jena University Hospital. *Nfatc2* ^−/−^ mice were maintained at the Zentrum für Experimentelle Molekulare Medizin (ZEMM), Würzburg, Germany. We used sex‐ and age‐matched wild‐type (wt), *Il1rl1* ^−/−^ and *Nfatc2* ^−/−^ [34] mice. Isolation of murine tissues was approved by the Thüringer Landesamt für Lebensmittelsicherheit und Verbraucherschutz (TLLV); Bad Langensalza. The licence for organ isolations at the Institute of Immunology, Jena is twz‐36‐2017.

### BMMC generation

Bone marrow was cultured in IMDM (PAA) (with 10%FCS, 100 U/ml penicillin, 100 mg/ml streptomycin, 50 mM β‐mercaptoethanol, 20 ng/ml X63Ag‐653 BPV‐rmIL‐3 supernatant, the source of IL‐3 for BMMC generation). Cells were used for experiments when cultures consisted of 95% BMMCs (after 4 weeks). BMMC purity was determined by analysis of the surface expression of c‐Kit and the FcεRI via flow cytometry.

### Isolation and expansion of peritoneal cavity derived MCs (PCMCs)

Peritoneal cavity cells were isolated by flushing the peritoneal cavity with PBS. Pooled peritoneal cells from 5 mice represent one biological sample. To get *n* = 3 biological replicates, we used 15 wt mice. Upon isolation, PCMCs were cultured in IMDM (PAA) supplemented with 10% FCS, 100 U/ml penicillin, 100 µg/ml streptomycin, 50 mM β‐mercaptoethanol and rmSCF (10 ng/ml) and rmIL‐3 (10 ng/ml) (PeproTech) for 14 days. The purity of c‐Kit^+^/FcεRI^+^ cells was analysed on day 14. MC cultures with a purity of 90–95% c‐Kit^+^/FcεRI^+^ cells were used for experiments.

### RNA sequencing and analysis

In the presence of IL‐3, BMMCs were stimulated with IL‐33 (both 50 ng/ml, from PeproTech) for 24 h. Subsequently, total RNA was isolated with Qiazol reagent (Qiagen). The sequence library was generated by introducing 200 ng of total RNA into the NEB Next Ultra II directional library preparation kit including enrichment of polyA RNAs using NEB Next Poly(A) mRNA Magnetic Isolation Module (both New England Biolabs). Libraries were sequenced using a NovaSeq6000 in 71 + 35 cycle paired‐end mode (SP 100 cycle kit, Xp workflow, Illumina). Reads were extracted in FastQ format using Illumina's bcl2fastq v2.20.0.422. Sequencing resulted in around 62 million reads per sample. For data analysis, only the forward mates (R1: 71bp) of the paired‐end reads were used. Mapping of reads was done to GRCm38.p6.92 using Top Hat (v2.1, parameters: ‐‐no‐convert‐bam‐‐no‐coverage‐search ‐x 1 ‐g 1) [35]. The reads per gene were counted using feature Counts (v1.6.3, parameters: ‐s 2) [36]. Counts (reads per gene) were analysed using the R package DESeq2 v1.26 [37] in order to find differentially expressed genes (DEG). For each gene, a *p*‐value was calculated using Wald significance test including correction for multiple testing following Benjamini and Hochberg. Differentially expressed genes were defined by *p* < 0·05 (10 938 genes). For GSE154104: FastQ files were downloaded from NCBI's SRA. Read pairs were adapter trimmed using cutadapt (v2.1, parameters: ‐q 10 ‐m 30). Mapping of read pairs, counting of reads pairs/fragments per gene and detection of DEGs was done similar as described above (except for featureCounts, here parameters ‐s 0 ‐p ‐B were set). DEGs in GSE154104 (DP0 vs. DP7) [25] were compared for intersection to genes listed in Dwyer *et al* [26]. In order to focus on highly regulated genes only those were introduced with a log2 (Fold Change) >2 and <−2. Twelve out of 82 genes from Dwyer *et al*. [26] are overlapping with 2001 up‐regulated genes (DP0 vs. DP7, *p* = 5·13*10^−5^, exact binominal test: R function ‘binom.test’). None of the 82 genes are overlapping with 88 down‐regulated genes (not significant).

### Stimulation of MCs

BMMCs, PCMCs or the MC/9‐NFAT and MC/9‐NF‐κB reporter MC lines ^38^ were washed and seeded in IL‐3‐free media at a density of 1x10^6^ cells/mL. After 1 h, cells were treated with cyclosporine A (4 µg/ml) (CaN inhibitor, CaN‐i), the Ca^2+^ chelator BAPTA‐AM (5 µM) (Ca^2+^ chel.) for Ca^2+^ depletion, the IKK VII (1 µM) (IKK inhibitor, IKK‐i), U0126 (10 µM) (ERK1/2 inhibitor, ERK‐i) (all from Merck), the selective P2X7 inhibitor, A438079 (50 µM) (ATP‐antagonist, ATP‐ant.) (Sigma), skepinone‐L (1 µM) (p38 inhibitor, p38‐i) (Cayman) or respective amounts of the vehicles (DMSO) for 30 min. All used inhibitors did not influence cell viability. Subsequently, cells were stimulated with rmIL‐33 (50 ng/mL) (PeproTech) and/ or if not otherwise shown with ATP (500 µM) (Sigma). For Western blotting experiments, BMMCs were stimulated for different time points (as shown in the figures). For ELISA experiments, supernatants were collected after 24 h, and for degranulation assays (CD107α surface expression), MCs were stimulated for 30 min. To investigate the activation of NFAT or NF‐κB in the MC/9 reporter MC lines [38], we stimulated for 8h.

### Ca^2+^ imaging

To determine the effect of ATP (Sigma) and/ or IL‐33 (50 ng/ml) (PeproTech) on the intracellular Ca^2+^ concentration [Ca^2+^]_I_, 5x10^6^ BMMCs were resuspended in KREPS‐HEPES buffer (20 mM HEPES pH 7·4,135 mM NaCl, 5 mM KCl, 1 mM MgSO_4_, 0·4 mM KH_2_PO_4_ and 5·5 mM glucose) and incubated with 0·2 µM Fura‐2/AM (Thermo Fisher Scientific, Waltham, MA) for 30min (37°celsius, 5% CO_2_). After centrifugation, cells were recovered in KREBS‐HEPES buffer supplemented with 1 mM CaCl_2_ and diluted to a final density of 1 × 10^6^ cells/ml. Cells were transferred onto black 96‐well microplates PS F‐bottom (Greiner bio‐one), and the signal was monitored by a NOVOstar microplate reader (BMG Labtechnologies GmbH) at 37°C : emission at 510 nm, excitation at 340 nm (Ca^2+^‐bound Fura‐2) and 380 nm (free Fura‐2). By subsequent cell lysis with triton X‐100, the maximal fluorescence signals were determined, followed by chelating Ca^2+^ with 20 mM EDTA to assess the minimal fluorescence.

### Flow cytometry

BMMCs were harvested and washed with PBA (PBS supplemented with 5 mg/mL BSA and 10 mM NaN_3_). BMMCs were treated with anti‐CD16/CD32 and rat‐IgG (Jackson) to prevent non‐specific binding of the staining antibodies. BMMCs were stained with PE‐anti‐CD117 or APC‐anti‐CD117, FITC‐anti‐FcεRI or PE‐CD107α (all Biolegend). MC/9‐NFAT and MC/9‐NF‐κB MC lines were stained with PE‐anti‐CD117. All cells were analysed with a LSR II flow cytometer (BD). Data were analysed with FlowJo 10 (Treestar Inc., Ashland, OR).

### Cell stimulation, lysis and immunoblotting

Washed BMMCs were seeded in IL‐3‐free media at a density of 10^6^ cells/ml. After 1 h, cells were stimulated with rmIL‐33 (50 ng/ml) (PeproTech) and/or ATP (500 µM) (Sigma). Lysis of the cells was performed with lysis buffer (20 mM HEPES, pH7·5; 10 mM EGTA, 40 mM β‐glycerophosphate, 2·5 mM MgCl_2_, 2 mM orthovanadate, 1 mM dithiothreitol, 20 µg/mL aprotinin and 20 µg/ml leupeptin supplemented with 1% Triton), and the protein concentration was determined (BCA‐kit; Pierce). Boiled samples (treated with 6 × Laemmli buffer) were separated on 10% sodium dodecyl sulphate (SDS)‐Laemmli gels. Gels were transferred onto nitrocellulose membranes (biostep) by electroblotting. After blocking (with dry milk), membranes were incubated with either anti‐pT180/pY182‐p38, anti‐pS177/181‐IKK2, anti‐p184/187‐pTAK1, anti‐pT202/pY204‐ERK1/2, anti‐pS32‐IκBα, and anti‐pS536‐p65 or the respective anti‐total antibodies (non‐phosphorylated proteins) [all from Cell Signaling except anti‐IKK1/2, anti‐ERK1/2 and anti‐tubulin (Santa Cruz)]. Washed membranes (TBS/0·1% Tween) were incubated with HRP‐conjugated secondary anti‐rabbit‐Ig, or anti‐mouse‐Ig or anti‐goat‐Ig (SeraCare). Detection was performed with ECL reagent (Pierce).

### LM metabololipidomics

BMMCs (1 × 10^6^ cells/ml) were seeded in IL‐3‐free media. After 1 h, cells were pre‐treated with the inhibitors cyclosporine (4 µg/ml) (CaN inhibitor, CaN‐i), the Ca^2+^ chelator BAPTA‐AM (5 µM) (Ca^2+^ chel.) for Ca^2+^ depletion, U0126 (10 µM) (ERK1/2 inhibitor, ERK‐i) (all from Merck), the selective P2X7 inhibitor, A438079 (50 µM) (ATP‐antagonist, ATP‐ant.) (Sigma) or skepinone‐L (1 µM) (Cayman) (p38 inhibitor, p38‐i) or vehicle for 30 min. Afterwards, BMMCs were stimulated with IL‐33 (50 ng/ml) (PeproTech) or ATP (500 µM) (Sigma) or both in combination for 24 h. Supernatants were transferred to ice‐cold methanol containing deuterium‐labelled internal standards (200 nM d8‐5S‐HETE, d4‐LTB_4_, d5‐LXA_4_, d5‐RvD2, d4‐PGE_2_ and 10 µM d8‐AA; Cayman) for the quantification of LM and sample recovery. Sample preparation was performed as recently shown recently reported [39]. In brief, proteins were precipitated, centrifuged and to the supernatant acidified H_2_O was added (1/9, v/v, final pH = 3·5). Samples were subjected to solid‐phase extraction using Sep‐Pak^®^ Vac 6cc 500 mg/6 ml C18 (Waters). After washing with H_2_O and n‐hexane, LM were eluted with methyl formate. Samples were dried using an evaporation system (TurboVap LV) and resuspended in methanol‐water (50/50, v/v) for UPLC‐MS‐MS automated injections. LM profiling was analysed with an Acquity™ UPLC system (Waters) and a QTRAP 5500 mass spectrometer (ABSciex) equipped with a TurboV™ Source and electrospray ionization. LM was eluted using an ACQUITY UPLC^®^ BEH C18 column (1·7 µm, 2·1 × 100 mm; Waters) at 50°C with a flow rate of 0·3 ml/min and a mobile phase consisting of methanol‐water‐acetic acid of 42:58:0·01 (v/v/v) that was ramped to 86:14:0·01 (v/v/v) over 12.5 min and then to 98:2:0·01 (v/v/v) for 3 min [40]. The QTrap 5500 was operated in negative ionization mode using scheduled multiple reaction monitoring (MRM) coupled with information‐dependent acquisition. The scheduled MRM window was 60 s, optimized LM parameters were adopted, and the curtain gas pressure was set to 35 psi. The retention time and at least six diagnostic ions for each LM were confirmed by means of an external standard (Cayman). Quantification was achieved by calibration curves for each LM. Linear calibration curves were obtained for each LM and gave r2 values of 0·998 or higher (for fatty acids 0·95 or higher). Additionally, the limit of detection for each targeted LM was determined.

### LTC_4_ ELISA

BMMCs (1 × 10^6^ cells/ml) were seeded in IL‐3‐ and serum‐free media. After 1 h, cells were pre‐treated with the inhibitors cyclosporine A (4 µg/ml) (CaN inhibitor, CaN‐i), the Ca^2+^ chelator BAPTA‐AM (5 µM) (Ca^2+^ chel.) for Ca^2+^ depletion, U0126 (10 µM) (ERK1/2 inhibitor, ERK‐i) (all from Merck) or skepinone‐L (1 µM) (Cayman) (p38 inhibitor, p38‐i) or vehicle for 30 min. Afterwards, BMMCs were stimulated with IL‐33 (50 ng/ml) (PeproTech) or ATP (500 µM) (Sigma) or both in combination for 24 h. Supernatants were transferred to pre‐coated ELISA plates and the competitive LTC_4_ ELISA was performed as described in the instructions (Cayman).

### Statistical analysis

All experiments were performed at least with three biological replicates. One biological replicate always is the bone marrow culture from at least 2 mice. Western blots intensities were determined with the ImageJ software (Fiji). The phospho‐specific Western blots were normalized to the total protein blots. The control (unstimulated sample) of wt BMMCs was set as 1. For analysis of Ca^2+^ imaging, the fold‐increase of measured intracellular Ca^2+^ [Ca^2+^]_I_ in nM of the mean of basal level against the mean of treatment level was calculated. For cytokine ELISA experiments biological replicates were split into at least a 4 technical replicates. For all experiments, significance was assessed by the Students *t*‐test. For LM analysis, Students' *t*‐test was performed with log‐transformed data (*p ≤ 0·05; **p ≤ 0·01; ***p ≤ 0·001). Statistics were performed with IBM SPSS Statistics version 20.0 (IBM, Ehningen, Germany).

## CONFLICT OF INTEREST

The authors declare no competing financial interests.

## AUTHOR CONTRIBUTIONS

P.M.J., N.A., M.G., O.W. and A. D. performed and analysed experiments, wrote and edited the manuscript; P.W., F.W., U.J. and C.K.: performed and analysed experiments, edited the manuscript; T.K. and E.S. provided material and edited the manuscript; S.D. developed the concept, designed the research, performed most of the experiments, analysed data, drafted, wrote and edited the paper.

## Supporting information

Fig S1

Fig S2

Fig S3

Fig S4

Fig S5

Fig S6

Fig S7

Fig S8

Fig S9

## Data Availability

The data discussed in this publication have been deposited in NCBI's Gene Expression Omnibus [41] and are accessible through GEO Series accession number GSE163065 (https://www.ncbi.nlm.nih.gov/geo/query/acc.cgi?acc=GSE163065). Data from Winkler et al (2020) were downloaded from NCBI's GEO/SRA (accession no. GSE154104).
