# Supplementary figures and images for: ATP/IL‐33‐triggered hyperactivation of mast cells results in an amplified production of pro‐inflammatory cytokines and eicosanoids
Source: Immunology. 2021 Jun 30;164(3):541–54. doi: 10.1111/imm.13386 (PMC8517600; doi:10.1111/imm.13386)

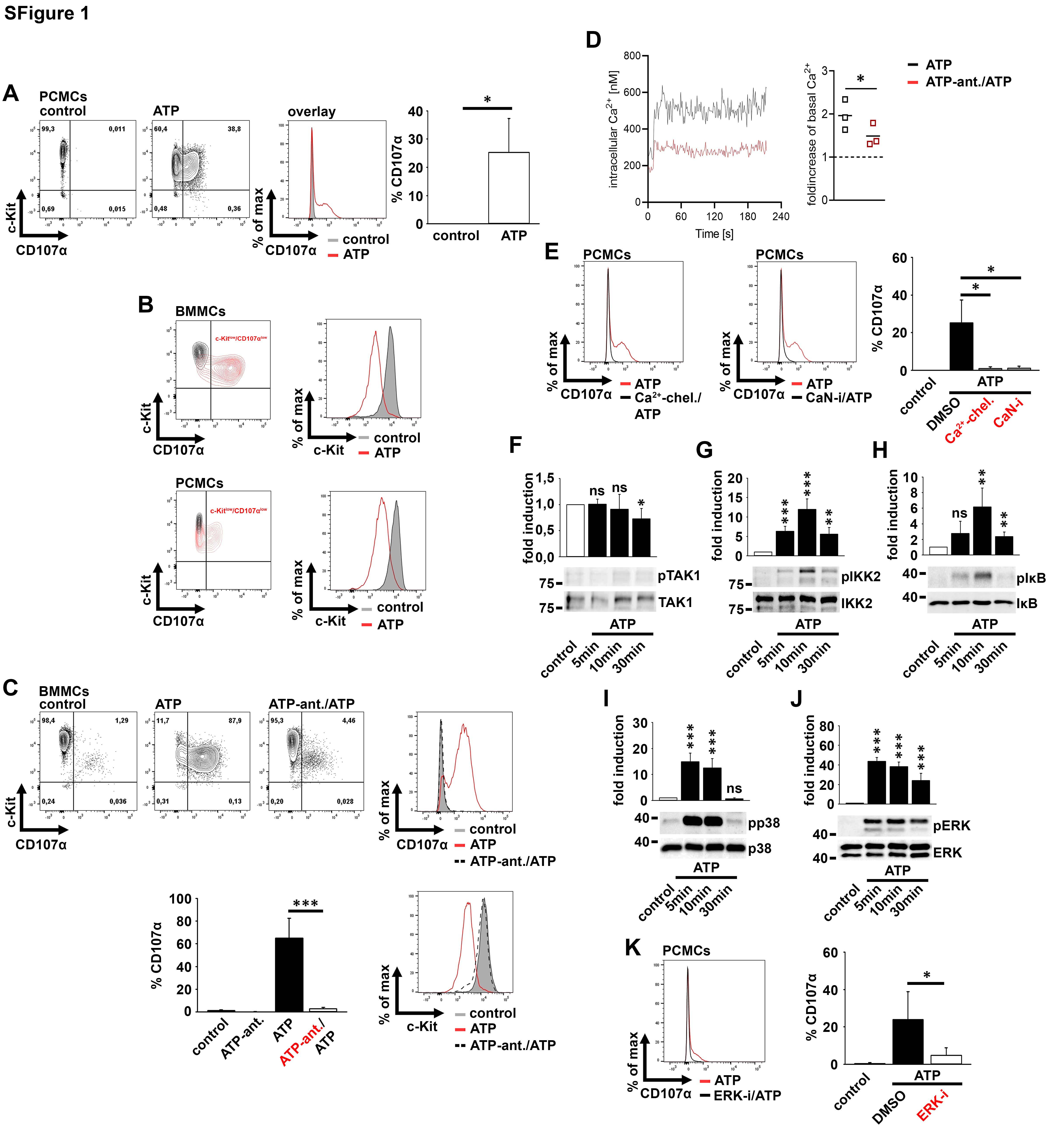

Supplement: Supplementary file 1 — Fig S1 [file IMM-164-541-s001.jpg]

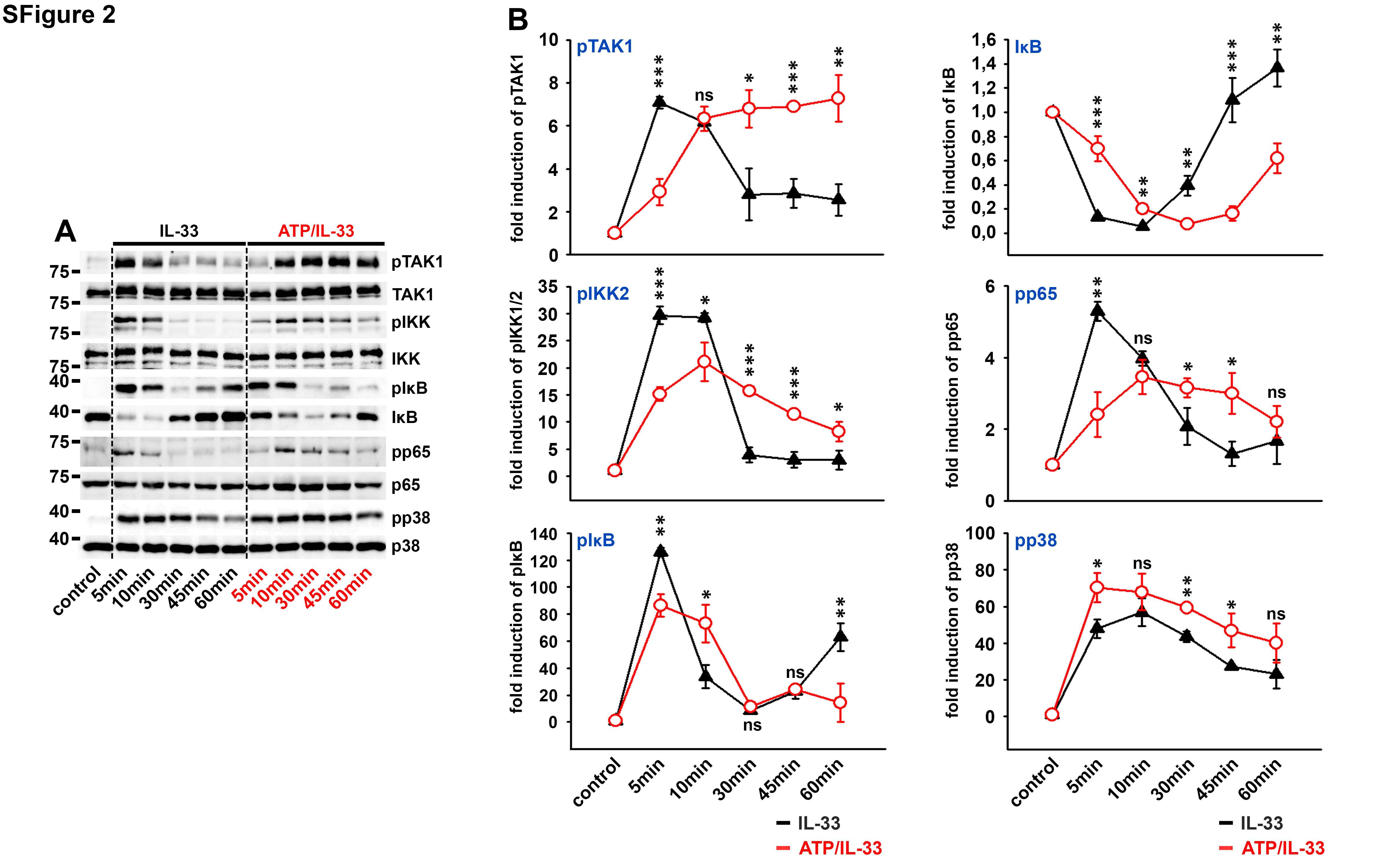

Supplement: Supplementary file 2 — Fig S2 [file IMM-164-541-s009.jpg]

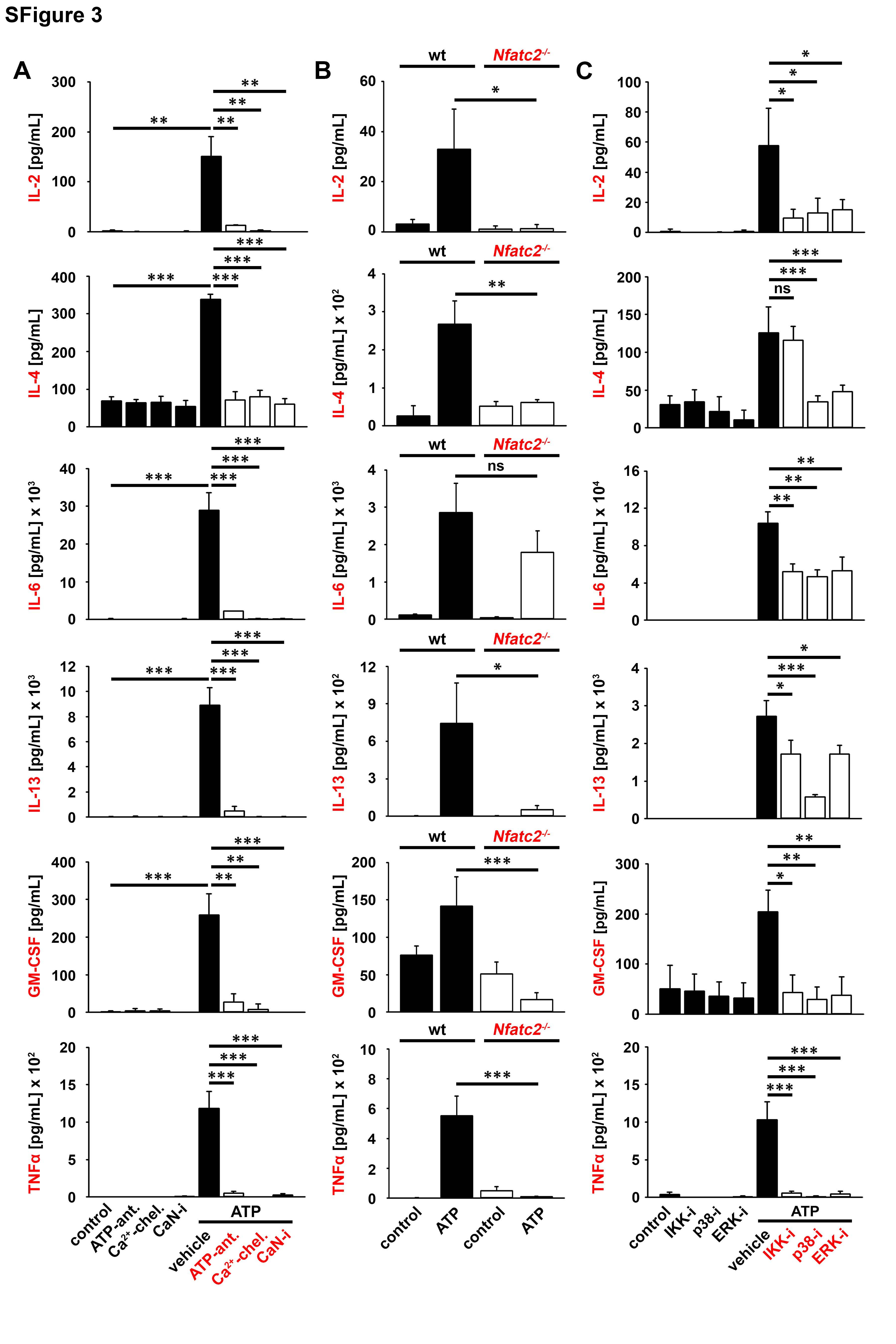

Supplement: Supplementary file 3 — Fig S3 [file IMM-164-541-s002.jpg]

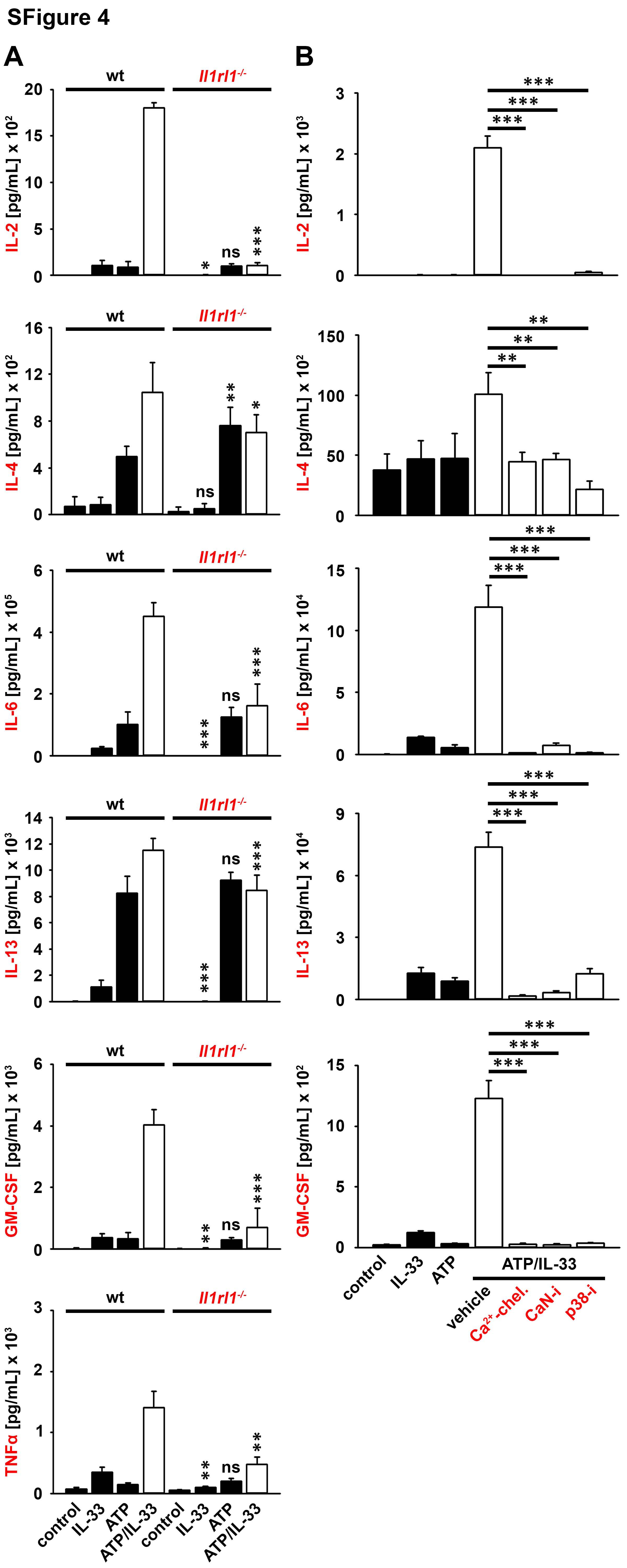

Supplement: Supplementary file 4 — Fig S4 [file IMM-164-541-s006.jpg]

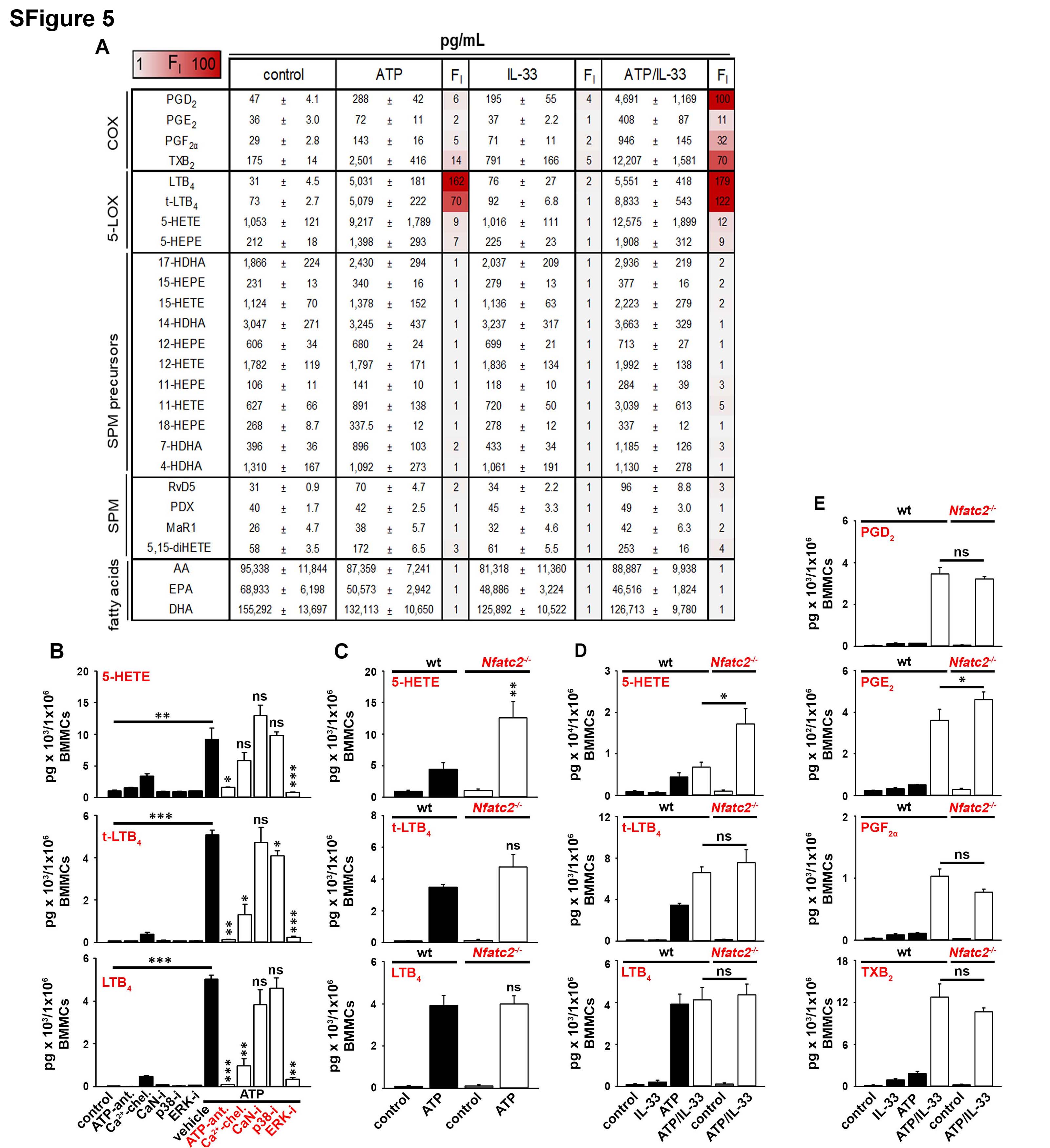

Supplement: Supplementary file 5 — Fig S5 [file IMM-164-541-s007.jpg]

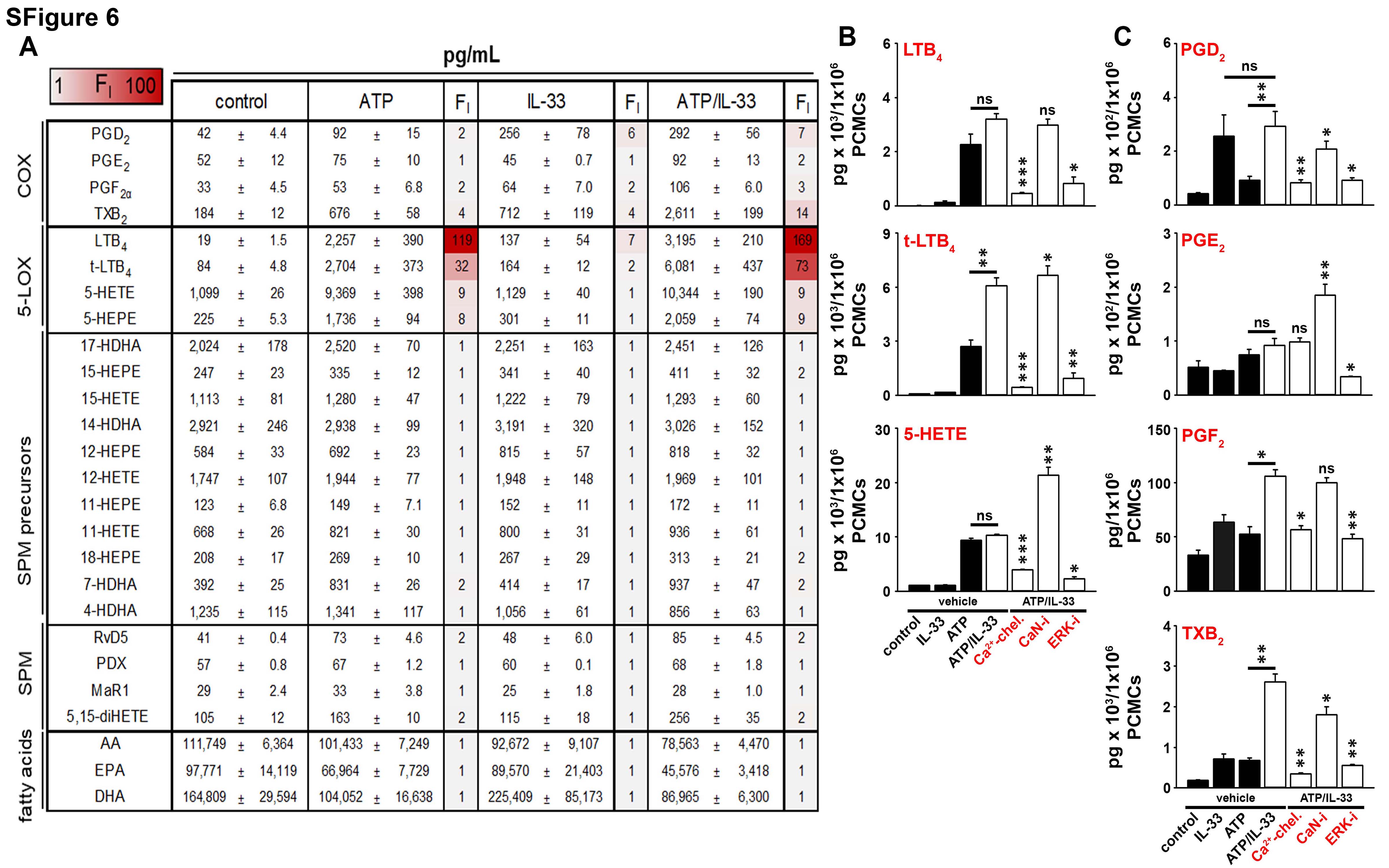

Supplement: Supplementary file 6 — Fig S6 [file IMM-164-541-s005.jpg]

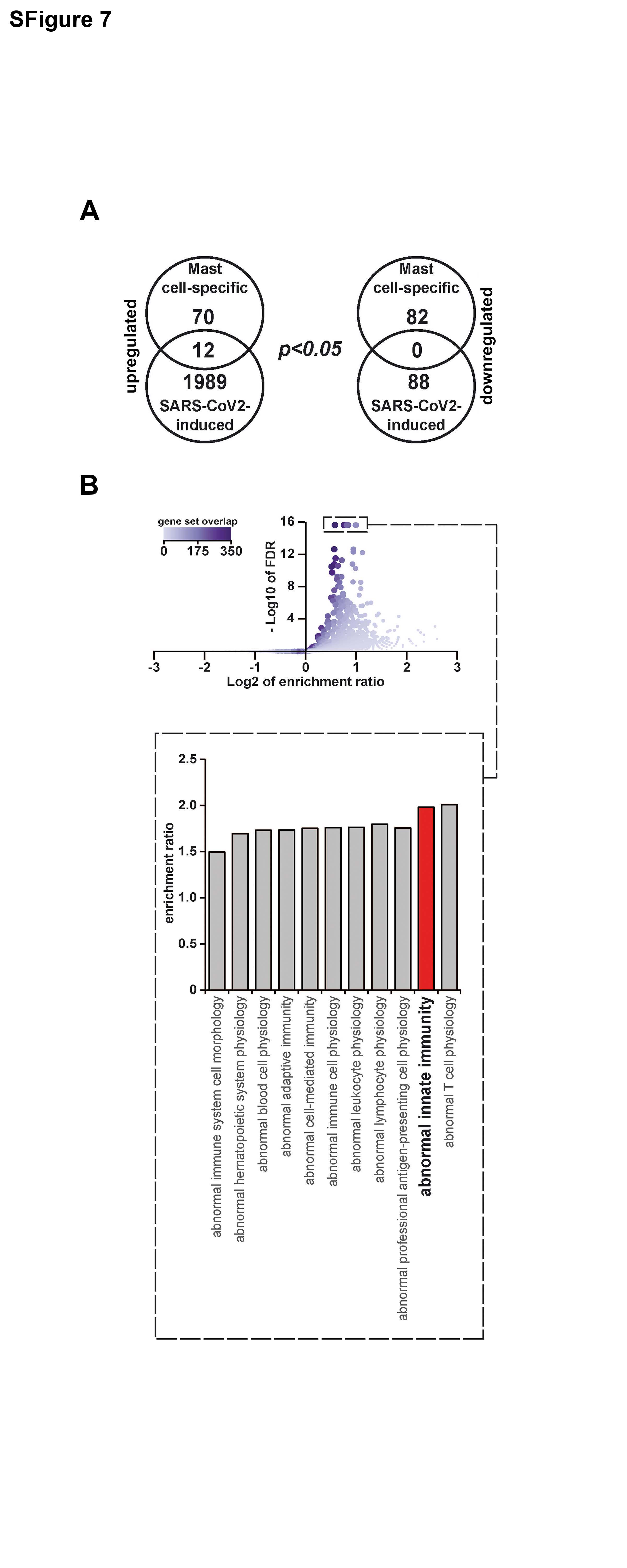

Supplement: Supplementary file 7 — Fig S7 [file IMM-164-541-s003.jpg]

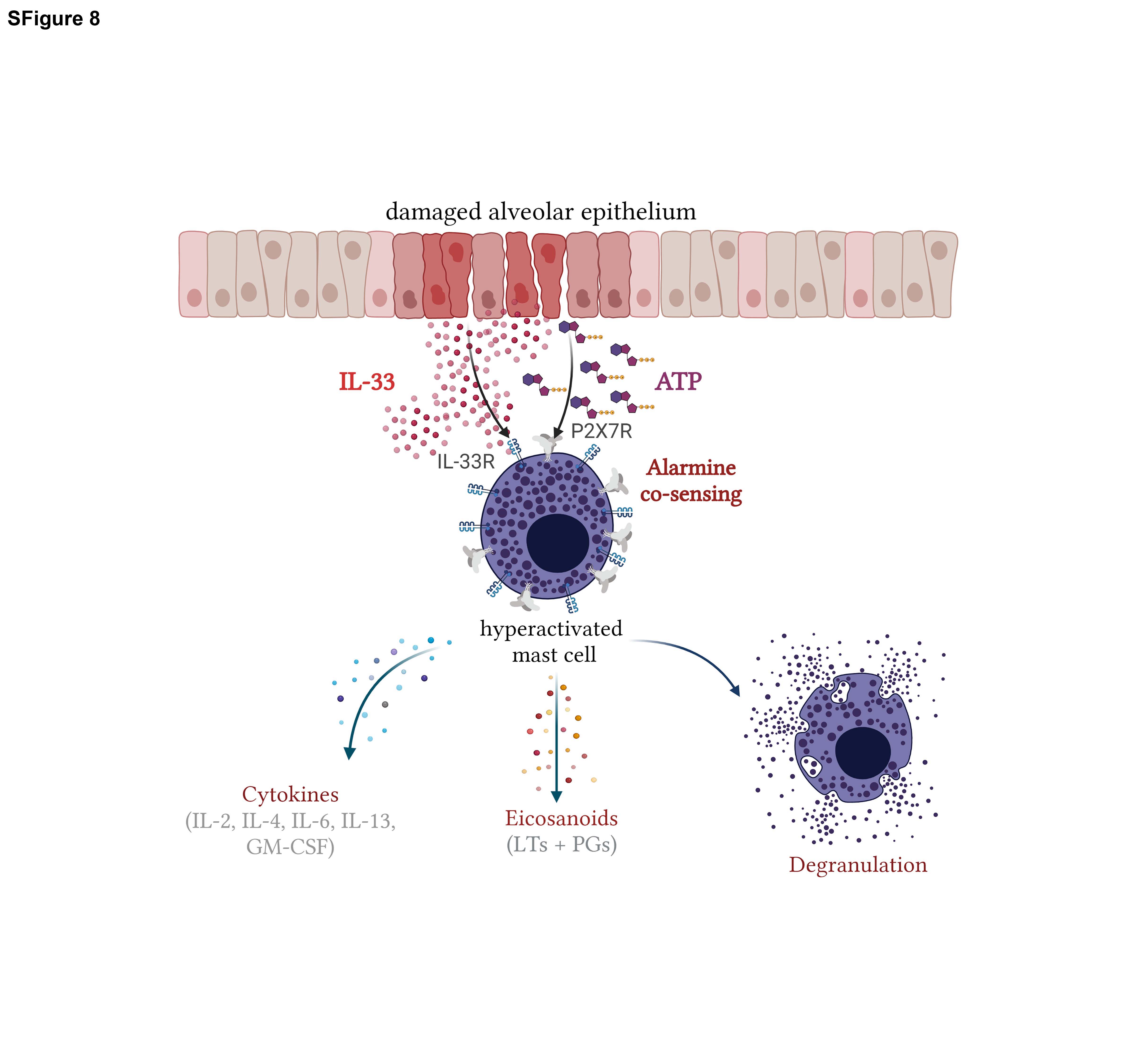

Supplement: Supplementary file 8 — Fig S8 [file IMM-164-541-s008.jpg]

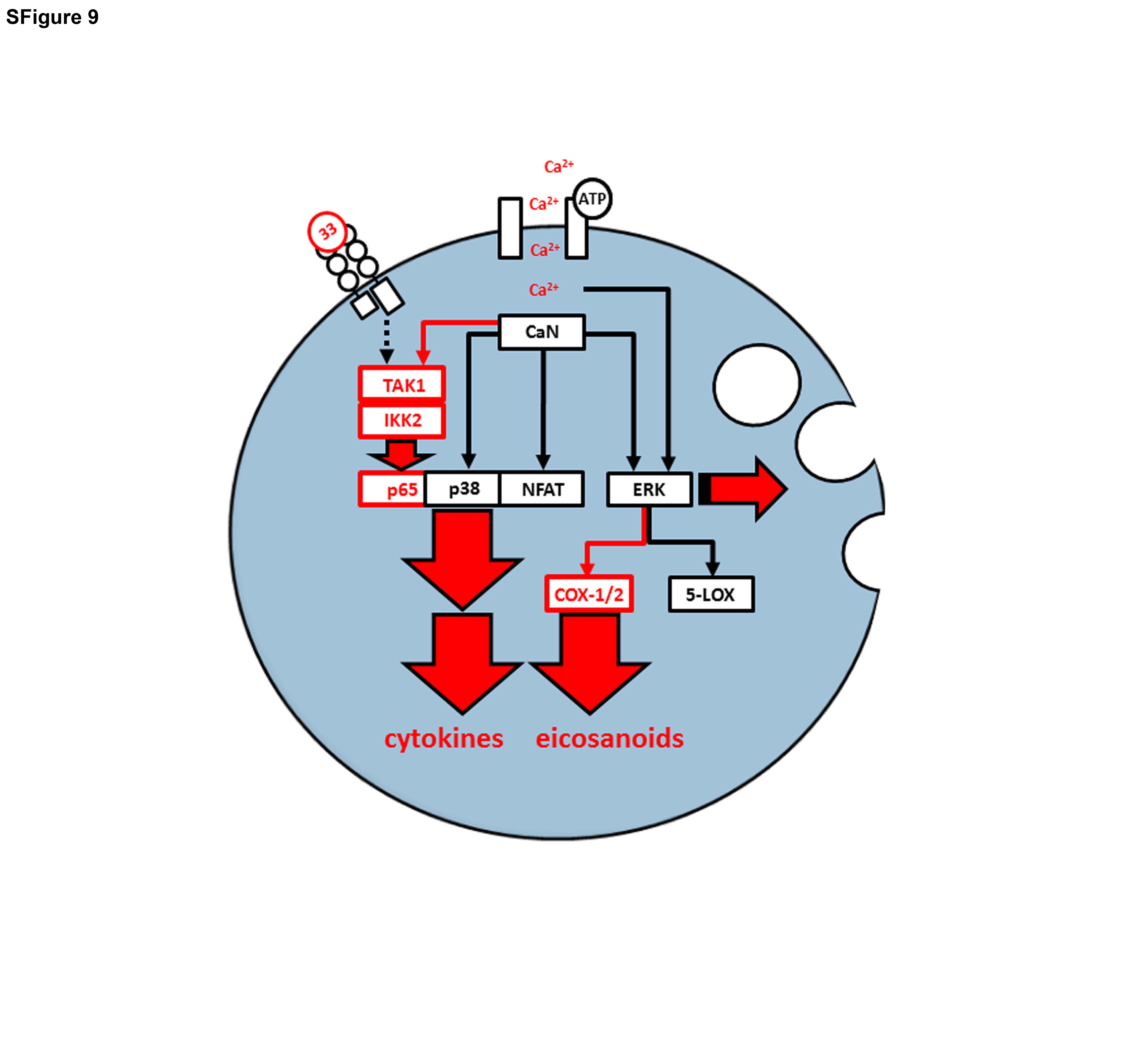

Supplement: Supplementary file 9 — Fig S9 [file IMM-164-541-s004.jpg]
